# Expansion of the known host range of *Giardia duodenalis* and *Cryptosporidium* spp. in captive wildlife at Beijing Zoo

**DOI:** 10.1051/parasite/2024079

**Published:** 2025-01-23

**Authors:** Qianming Zhao, Zhiyang Pei, Yongqiang He, Ting Jia, Yanzhen Zhang, Mengjun Zheng, Zhenjie Zhang, Meng Qi

**Affiliations:** 1 College of Animal Science and Technology, Tarim University Alaer Xinjiang 843300 China; 2 Beijing Zoo, Beijing Key Laboratory of Captive Wildlife Technologies Beijing 100044 China

**Keywords:** *Giardia duodenalis*, *Cryptosporidium* spp., Zoonotic, Wild animals, Host

## Abstract

*Giardia duodenalis* and *Cryptosporidium* spp. are significant zoonotic parasites that cause diarrhea and affect a diverse range of hosts. This study aimed to investigate the infection rates of these parasites in captive wildlife at Beijing Zoo. A total of 445 fecal samples were collected and analyzed using PCR. The infection rate of *G. duodenalis* was 3.82% (17/445), with assemblage A (*n* = 1), assemblage B (*n* = 13), and assemblage E (*n* = 3) identified. The infection rate of *Cryptosporidium* spp. was 0.22% (1/445), with only one instance of *C. ryanae* identified from cattle (*Bos taurus*). To the best of our knowledge, this study enhances the understanding of the host range of *G. duodenalis* in captive wildlife within China, highlighting infections in Corsac Fox (*Vulpes corsac*), Indian Rhinoceros (*Rhinoceros unicornis*), and Giraffe (*Giraffa camelopardalis*). These findings indicate that the infection rates of *G. duodenalis* and *Cryptosporidium* spp. in captive wildlife at Beijing Zoo are low, while showing that the known host range of *G. duodenalis* is expanding.

## Abstract (fr)

*Giardia duodenalis* et *Cryptosporidium* spp. sont des parasites zoonotiques importants qui provoquent des diarrhées et affectent une gamme diversifiée d’hôtes. Cette étude visait à examiner les taux d’infection de ces parasites chez la faune captive au zoo de Pékin. Au total, 445 échantillons fécaux ont été collectés et analysés par PCR. Le taux d’infection de *G. duodenalis* était de 3,82 % (17/445), avec l’assemblage A (*n* = 1), l’assemblage B (*n* = 13) et l’assemblage E (*n* = 3) identifiés. Le taux d’infection de *Cryptosporidium* spp. était de 0,22 % (1/445), avec un seul cas de *C. ryanae* identifié chez des bovins (*Bos taurus*). À notre connaissance, cette étude améliore la compréhension de la gamme d’hôtes de *G. duodenalis* chez la faune captive en Chine, mettant en évidence des infections chez le renard corsac (*Vulpes corsac*), le rhinocéros indien (*Rhinoceros unicornis*) et la girafe (*Giraffa camelopardalis*). Ces résultats indiquent que les taux d’infection de *G. duodenalis* et de *Cryptosporidium* spp. dans la faune captive du zoo de Pékin sont faibles, tout en montrant que la gamme d’hôtes connue de *G. duodenalis* est en expansion.

## Introduction

*Cryptosporidium* spp. and *Giardia duodenalis* are prevalent and significant intestinal protozoan parasites that cause clinical manifestations such as diarrhea, dehydration, and anorexia in a diverse array of vertebrate hosts, including humans [2, 24]. Currently, at least 47 species and 120 genotypes of *Cryptosporidium* spp. have been identified, with 19 species known to infect humans [10, 14, 21, 24, 29], underscoring the potential for zoonotic transmission. *Giardia duodenalis* comprises eight assemblage types (A–H) [5, 26], with assemblages A and B capable of infecting a wide range of mammals, including humans, while assemblages C–H exhibit more stringent host specificity [4, 23]. Notably, human infections with assemblages C, E, and F have also been documented [1, 27, 28].

Beijing Zoo, a national zoo covering an area of 80.5 hectares (approximately 199 acres), is home to around 400 species of wild animals, totalling more than 5,000 individual animals. As a vital component of the ecosystem, wild animals play a significant role in the biosphere. Zoos are essential for the conservation of wild animal populations and the preservation of species diversity. However, the spatial constraints of zoos differ markedly from the conditions of free-ranging life in the wild, leading to a higher prevalence of parasites among captive wildlife compared to their naturally free-ranging counterparts [19]. Animals harboring pathogens not only pose health risks to their own populations, but also contribute to the transmission of zoonotic diseases through close interactions with keepers and visitors [8].

The objective of this study was to characterize the epidemiology of *G. duodenalis* and *Cryptosporidium* spp. in captive wildlife at the Beijing Zoo. This research aims to identify new host species and provide valuable insights for biological and epidemiological studies of these significant zoonotic parasites.

## Materials and methods

### Ethics declarations

The protocol for this study did not require review and approval from the Animal Ethics Committee. Prior permission was obtained from the zoo management before collecting fecal samples. The fecal samples were systematically collected from the floor, ensuring that the animals were not harmed.

### Fecal sample collection

In September 2020, a total of 445 wildlife fecal samples were collected from Beijing Zoo, consisting of 322 samples from mammals and 123 samples from birds (see Supplementary Table 1). Mammals were sampled individually by keepers to obtain freshly excreted feces, with the understanding that this sampling is a one-time event with no repeat sampling. For birds, endangered and rare species (e.g., black stork, red-crowned cranes) and specific parrots (e.g., Amazon parrots, macaws) were also sampled individually. In contrast, flock groups of small birds (e.g., flamingos, white-naped cranes, grey cranes) and passeriformes were sampled collectively, with fresh fecal samples randomly selected from the ground. It is important to note that the data collection process did not accurately reflect the sex and age of each animal.

All animals are classified and housed according to their respective species. Each specific animal resides in a well-ventilated and sunny area, ensuring that contact with animals of different species is prevented. Most animals are provided with food through feeding troughs located within their enclosures, and they either share drinking troughs or utilize centralized water supply facilities. The environmental setup of the enclosures includes areas visible to visitors as well as sections managed by keepers. The extent of direct human contact varies among species; for instance, primates are typically isolated from visitors, while keepers have more frequent interactions with them. Importantly, the animals in this zoo had not been tested for *Giardia* prior to the commencement of our study.

Samples were collected in sterile sampling bags or boxes, with individual weights ranging from 1 to 20 g. Each sample was assigned a unique number and registered alongside the corresponding animal species and other relevant information. Subsequently, the samples were stored in a refrigerator at 4 °C and DNA extraction was conducted within 72 h.

### DNA extraction and PCR amplification

Fecal DNA was extracted using an E.Z.N.A.^®^ Stool DNA Kit (OMEGA Bio-tek Inc., Norcross, GA, USA). Approximately 200 mg of the fecal sample was placed in a 2 mL centrifuge tube, resulting in a final volume of approximately 200 μL of the extracted DNA sample.

The small subunit ribosomal RNA (*SSU* rRNA) gene (587 bp) was utilized for the detection of *Cryptosporidium* spp. [25]. Similarly, the *SSU* rRNA gene (290 bp) was employed for the detection of *G. duodenalis* [3]. Regarding samples identified as positive for *G. duodenalis*, a subsequent multilocus analysis was conducted using the *bg*, *gdh*, and *tpi* loci [15]. All PCR amplifications were performed using the 2× EasyTaq PCR SuperMix (TransGene Biotech Co., Beijing, China). The final PCR amplicons were visualized by electrophoresis on 1% agarose gels stained with Golden View (Sangon, Shanghai, China), utilizing 5 μL of each sample.

### Sequence analysis and phylogenetic tree construction

All secondary PCR-positive products were sequenced by GENEWIZ (Suzhou, China). The resulting sequences were uploaded to DNAStar’s SEQMAN (http://www.dnastar.com/) for proofreading of the DNA profiles, and were subsequently compared and identified using Clustal X2.1 (http://www.clustal.org/) and GenBank (https://www.ncbi.nlm.nih.gov/genbank/). A phylogenetic tree of *SSU* rRNA from *G. duodenalis* was constructed using the neighbor-joining method in MEGA 7.0 software. The reliability of the constructed trees was evaluated through bootstrap analysis with 1,000 replications.

### Nucleotide sequence accession numbers

The nucleotide sequences generated in this study have been submitted to the National Center for Biotechnology Information (NCBI) GenBank database. The accession numbers for the *G. duodenalis* 16s locus are OR141890 and PP987858–PP987865. For the *bg* locus, the accession numbers are PQ614254–PQ614256, as well as PQ614259 and PQ614260. The *gdh* locus is represented by the accession number PQ614263, while the *tpi* locus has accession numbers range from PQ614263 to PQ614268. Additionally, the accession number for *C. ryanae* is PP989415.

## Results

### Detection of *Giardia duodenalis* and *Cryptosporidium* spp.

An analysis of 445 collected samples revealed that 4.04% (18/445) tested positive for parasites. Among these, the prevalence of *G. duodenalis* was 3.82% (17/445), while *Cryptosporidium* spp. accounted for 0.22% (1/445). All positive samples originated from mammals, and no instances of mixed infections with both parasites were observed in the same animal. Furthermore, no parasitic infections were detected in ornithischians.

Primates of the family Lemuridae exhibited the highest overall prevalence of parasites, recorded at 47.06% (8/17). The parasites infecting these primates were exclusively *G. duodenalis*, which was found in both the black-and-white ruffed lemur (*n* = 4) and the ring-tailed lemur (*n* = 4) (refer to Supplementary Table 1). Furthermore, *G. duodenalis* infections were also identified in members of the Corsac fox (Canidae), polar bear (Ursidae), masked palm civet (Viverridae), and giraffe (Giraffidae). Additionally, *Cryptosporidium* spp. was detected in a single sample from cattle (*Bos taurus*).

### Genotyping in wild animal fecal samples

Assemblage typing was performed on 17 positive samples of *G. duodenalis*, revealing three distinct assemblage types: assemblage A (*n* = 1), assemblage B (*n* = 13), and assemblage E (*n* = 3) (see Table 1). Amplification of positive samples based on the *bg*, *gdh*, and *tpi* loci yielded a total of eight sequences at the *bg* locus (assemblage A, *n* = 1; assemblage B, *n* = 5; assemblage E, *n* = 2); six sequences at the *tpi* locus (assemblage A, *n* = 1; assemblage B, *n* = 5), and only one sequence of assemblage E at the *gdh* locus (Table 2).

**Table 1:** Occurrence of *G. duodenalis* and *Cryptosporidium* spp. in wildlife at Beijing Zoo.

Order	Family	Sample (*n*)	*Cryptosporidium* spp.		*G. duodenalis*	
			Prevalence (%)	Specie (*n*)	Prevalence (%)	Assemblage (*n*)
Primates	Cercopithecidae	63	–	–	–	–
	Lemuridae	13	–	–	47.06	B (8)
	Hylobatidae	12	–	–	–	–
	Aotidae	6	–	–	–	–
	Lorisidae	6	–	–	–	–
	Pongidae	3	–	–	–	–
	Atelidae	2	–	–	–	–
Carnivora	Felidae	24	–	–	–	–
	Ursidae	22	–	–	5.88	A (1)
	Canidae	13	–	–	5.88	B (1)
	Procyonidae	12	–	–	–	–
	Mustelidae	5	–	–	–	–
	Herpestidae	4	–	–	–	–
	Viverridae	2	–	–	5.88	B (1)
Artiodactyla	Bovidae	37	100	*C. ryanae* (1)	–	–
	Cervidae	23	–	–	–	–
	Giraffidae	7	–	–	17.64	E (3)
	Hippopotamidae	1	–	–	–	–
Perissodactyla	Equidae	17	–	–	–	–
	Tapiridae	16	–	–	–	–
	Rhinocerotidae	6	–	–	5.88	B (1)
Proboscidea	Elephantidae	8	–	–	–	–
Diprotodontia	Macropodidae	7	–	–	–	–
Rodentia	Caviidae	4	–	–	11.76	B (2)
	Sciuridae	1	–	–	–	–
	Hystricidae	1	–	–	–	–
Pilosa	Bradypodidae	4	–	–	–	–
	Myrmecophagidae	3	–	–	–	–
Gruiformes	Gruidae	49	–	–	–	–
Galliformes	Phasianidae	14	–	–	–	–
	Cracidae	1	–	–	–	–
Psittaciformes	Psittaculidae	6	–	–	–	–
	Psittacidae	4	–	–	–	–
	Cacatuidae	1	–	–	–	–
Bucerotiformes	Bucerotidae	5	–	–	–	–
	Bucorvidae	1	–	–	–	–
Passeriformes	Estrildidae	3	–	–	–	–
	Corvidae	2	–	–	–	–
	Alaudidae	1	–	–	–	–
	Sturnidae	1	–	–	–	–
Strigiformes	Strigidae	1	–	–	–	–
Piciformes	Ramphastidae	1	–	–	–	–
Anseriformes	Anatidae	15	–	–	–	–
Phoenicopteriformes	Phoenicopteridae	6	–	–	–	–
Ciconiiformes	Ciconiidae	3	–	–	–	–
Pelecaniformes	Ardeidae	2	–	–	–	–
Struthioniformes	Casuariidae	5	–	–	–	–
Charadriiformes	Laridae	1	–	–	–	–
Cuculiformes	Cuculidae	1	–	–	–	–
Total		445	0.22	*C. ryanae* (1)	3.82	A (1); B (13); E (3)

**Table 2:** Amplification results of different loci in *Giardia duodenalis*-positive samples.

Gene locus	Assemblage	Common name (Scientific name)	Positive samples (No.)	Accession number
*bg*	A (*n* = 1)	Polar Bear (*Ursu*s *maritimus*)	1	PQ614254
	B (*n* = 5)	Black-and-white Ruffed Lemur (*Varecia variegata*)	2	PQ614255
		Ring-tailed Lemur (*Lemur catta*)	2	PQ614256
		Corsac Fox (*Vulpes corsac*)	1	PQ614259
		Patagonian Mara (*Dolichotis patagonum*)	1	PQ614260
	E (*n* = 2)	Giraffe (*Giraffa camelopardalis*)	1	PQ614261
		Giraffe (*Giraffa camelopardalis*)	1	PQ614262
*gdh*	E (*n* = 1)	Giraffe (*Giraffa camelopardalis*)	1	PQ614263
*tpi*	A (*n* = 1)	Polar Bear (*Ursu*s *maritimus*)	1	PQ614264
	B (*n* = 5)	Ring-tailed lemur (*Lemur catta*)	2	PQ614265
		Ring-tailed lemur (*Lemur catta*)	1	PQ614267
		Black-and-white Ruffed Lemur (*Varecia variegata*)	1	PQ614266
		Domesticated Guinea Pig (*Cavia porcellus*)	1	PQ614268

**Figure 1:**
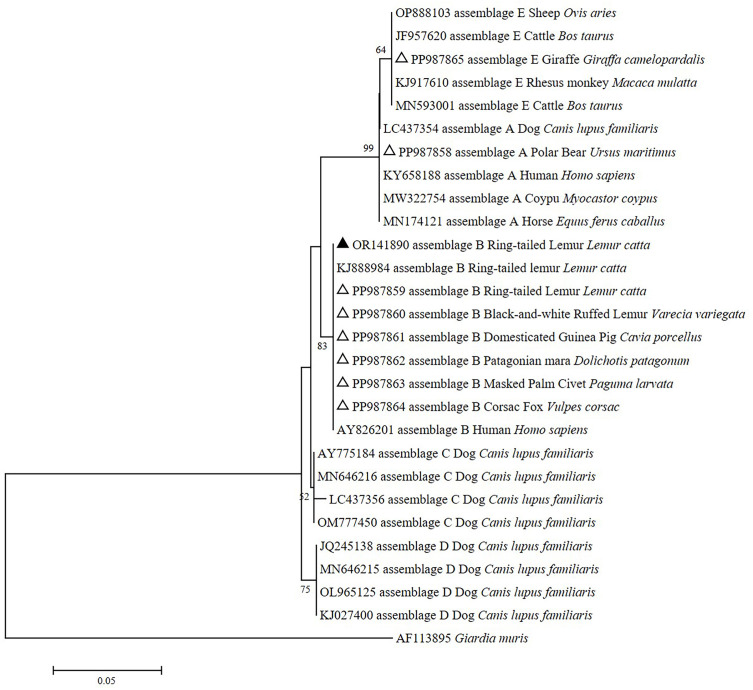
Phylogenetic analysis of *Giardia duodenalis* conducted based on small subunit (*SSU*) rRNA gene sequences. The evolutionary history was inferred using the neighbor-joining method, with analyses performed in MEGA 7.0. Bootstrap values greater than 50% from 1,000 replicates are indicated on the nodes. The sequences identified in this study are represented by triangles; known sequences are marked with open triangles, while new sequences are indicated by filled triangles.

**Figure 2:**
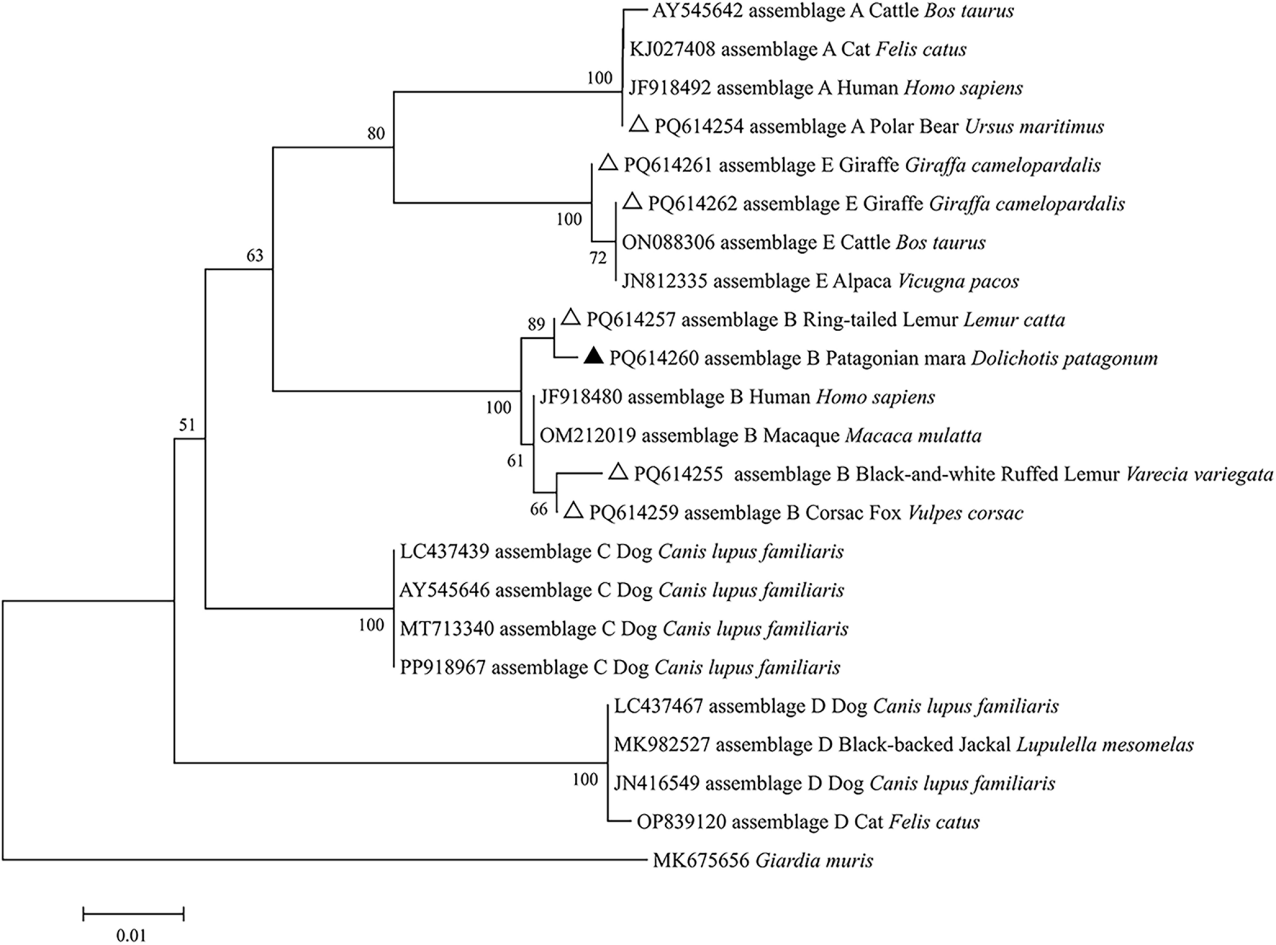
Phylogenetic analysis of *Giardia duodenalis* conducted based on beta-giardin (*bg*) gene sequences. The evolutionary history was inferred using the neighbor-joining method, with analyses performed in MEGA 7.0. Bootstrap values greater than 50% from 1,000 replicates are indicated on the nodes. The sequences identified in this study are represented by triangles; known sequences are marked with open triangles, while new sequences are indicated by filled triangles.

Additionally, relationships were observed between animal families and *G. duodenalis* assemblages. For example, samples from the families Rhinocerotidae, Caviidae, Lemuridae, Canidae, and Viverridae were classified under assemblage B. Notably, only one instance of assemblage A was identified in samples from the polar bear (Ursidae), while three samples from the giraffe (Giraffidae) were classified as assemblage E (Table 3). Based on the *SSU* rRNA locus, the newly obtained *G. duodenalis* assemblage B sequences in this study exhibited high alignment with the GenBank reference sequences and clustered with *G. duodenalis* assemblage B isolates from horses, humans and dogs (Fig. 1). Similarly, based on the *bg* locus, the newly obtained *G. duodenalis* assemblage B sequences were also highly consistent with the GenBank reference sequences, clustering with *G. duodenalis* assemblage B isolates from humans and macaques (Fig. 2). One *Cryptosporidium*-positive sample was genotyped and identified as *C. ryanae* (Table 1), with accession number PP989415, demonstrating 100% homology to the sequence MT374189 of a previously identified calf-derived isolate (Table 3).

**Table 3:** Distribution of wildlife *Giardia duodenalis* and *Cryptosporidium* spp. and their *SSU* rRNA gene sequences in Beijing Zoo.

Species/Assemblage	Common name (Scientific name)	Positive samples (No.)	Accession number
*Cryptosporidium ryanae*	Cattle (*Bos taurus*)	1	PP989415
*Giardia duodenalis* A	Polar Bear (*Ursu*s *maritimus*)	1	PP987858
*Giardia duodenalis* B	Ring-tailed Lemur (*Lemur catta*)	4	PP987859
	Black-and-white Ruffed Lemur (*Varecia variegata*)	4	PP987860
	Domesticated Guinea Pig (*Cavia porcellus*)	1	PP987861
	Patagonian Mara (*Dolichotis patagonum*)	1	PP987862
	Masked Palm Civet (*Paguma larvata*)	1	PP987863
	Corsac Fox (*Vulpes corsac*)	1	PP987864
	Indian Rhinoceros (*Allomyrina dithotomus*)	1	**OR141890**
*Giardia duodenalis* E	Giraffe (*Giraffa camelopardalis*)	3	PP987865

Bold: newly obtained sequences from this study.

## Discussion

*Giardia duodenalis* and *Cryptosporidium* spp., both significant zoonotic parasitic protozoa, have been reported globally in various captive and wild animal populations [12, 26, 31]. In this study, the infection rate of *G. duodenalis* was found to be 3.82% (17/445), which is lower than the rates reported in zoos across three cities in China: 10.57% (71/672) in Hangzhou, Dalian, and Suzhou [34], and 39.13% (9/23) in captive wildlife in central Colombia [6]. Conversely, this rate is higher than the infection rates observed at Zhengzhou Zoo, which reported 2.46% (5/203) [17] and in six zoos in Henan, where the infection rate was 0.47% (2/429) [33]. The infection rate for *Cryptosporidium* spp. was recorded at 0.22% (1/445), which is lower than the 8.19% (19/232) found in a zoo in Anhui [13] and 3.5% (7/200) in the National Zoo of Bangladesh [15], but higher than the 0.00% (0/116) reported in three zoos in Spain [16]. These findings illustrate a global variation in the prevalence of *G. duodenalis* and *Cryptosporidium* spp. among captive wildlife.

*Giardia duodenalis* cysts and *Cryptosporidium* spp. oocysts can persist for extended periods in water and soil [30]. Given that mammals primarily inhabit terrestrial environments, they are more likely to encounter infectious cysts and oocysts compared to avian species. This may partially explain the absence of detected parasites in the avifauna within this study. Additionally, primates, which exhibit more complex social behaviors [22], may experience increased parasite transmission due to direct contact and group living. Similarly, rodents, which are also social animals, have a higher prevalence of parasitic infections, suggesting that frequent contact among animals facilitates the transmission of intestinal parasites (Supplementary Table 1).

This study identified three assemblage types of *G. duodenalis* (Assemblages A, B, and E) among 17 positive samples. Assemblages A and B are the two most prevalent types of *G. duodenalis* that infect humans. These assemblages have also been detected in livestock, companion animals, and non-human primates [12]. Among the analyzed samples, Assemblage B was the most frequently observed type, identified in specimens from the families Lemuridae, Canidae, Viverridae, Rhinocerotidae, and Caviidae. This diverse representation across various mammalian groups further supports the notion that Assemblage B can infect a wide range of mammals [12]. In contrast, Assemblage E typically infects artiodactyls and is generally regarded as host-specific [4]; however, there have been documented cases of human infections with Assemblage E [9]. Consequently, the data suggest that zoo animals may serve as reservoir hosts for *G. duodenalis*, indicating the potential for cross-species transmission. Nonetheless, this hypothesis requires further research and additional data for validation.

Previous studies have demonstrated that *C. ryanae* primarily infects bovids, including cattle, yaks, and water buffaloes [11]. This study identified an isolate of *C. ryanae* in cattle, consistent with earlier research findings. However, some investigations have indicated that *C. ryanae* is also capable of infecting non-bovid animals, such as marsh deer (*Blastocerus dichotomus*) [20], horses (*Equus ferus caballus*) [18], sika deer (*Cervus nippon centralis*) [32], red deer (*Cervus elaphus*), roe deer (*Capreolus capreolus*), and wild boar (*Sus scrofa*) [7]. The ability of *C. ryanae* to infect various non-bovid hosts suggests that certain environmental and ecological factors may facilitate its transmission across species. Understanding these factors could enhance our knowledge of the epidemiology and control of cryptosporidiosis in both domestic and wild animal populations.

This study represents the inaugural report of *G. duodenalis* infections in Canidae (specifically, the Corsac Fox) and Indian Rhinoceros (Rhinocerotidae). The identification of these new hosts broadens the established host range of *G. duodenalis*, indicating that the parasite’s prevalence in wildlife may be more widespread than previously acknowledged. This finding underscores the need for an investigation into the potential roles of human activities, including zoo management practices, in facilitating the contamination of captive animals with zoonotic parasites. However, our investigation does not provide conclusive evidence regarding whether these animals serve as natural hosts or merely as carriers of the pathogen, nor does it clarify whether *G. duodenalis* can induce disease in these newly identified hosts. Additional research is essential to elucidate its pathological effects and transmission pathways, which will aid in the development of more effective control strategies.

## Conclusions

The findings of this study demonstrate the presence of *Cryptosporidium* spp. and *G. duodenalis* infections in captive wild animals at Beijing Zoo. These results expand the known host range of *G. duodenalis* and provide valuable insights into the epidemiology of these parasites. This information is crucial for the development of effective control and prevention strategies for both wildlife and domestic animals.

## Supplementary material

The supplementary material for this article can be found at https://www.parasite-journal.org/10.1051/parasite/2024079/olm. *Supplementary Table 1*: List of specimens studied and results for *Giardia duodenalis* and *Cryptosporidium* spp.
